# Behavioral and Electrophysiological Characterization of *Dyt1* Heterozygous Knockout Mice

**DOI:** 10.1371/journal.pone.0120916

**Published:** 2015-03-23

**Authors:** Fumiaki Yokoi, Huan-Xin Chen, Mai Tu Dang, Chad C. Cheetham, Susan L. Campbell, Steven N. Roper, J. David Sweatt, Yuqing Li

**Affiliations:** 1 Department of Neurology, College of Medicine, University of Florida, Gainesville, Florida, United States of America; 2 Children’s Hospital of Philadelphia, Philadelphia, Pennsylvania, United States of America; 3 Center for Neurodegeneration and Experimental Therapeutics, Department of Neurology, School of Medicine, University of Alabama at Birmingham, Birmingham, Alabama, United States of America; 4 Department of Neurobiology, School of Medicine, University of Alabama at Birmingham, Birmingham, Alabama, United States of America; 5 Department of Neurosurgery, College of Medicine, University of Florida, Gainesville, Florida, United States of America; University of Chicago, UNITED STATES

## Abstract

DYT1 dystonia is an inherited movement disorder caused by mutations in *DYT1* (*TOR1A*), which codes for torsinA. Most of the patients have a trinucleotide deletion (ΔGAG) corresponding to a glutamic acid in the C-terminal region (torsinA^ΔE^). *Dyt1* ΔGAG heterozygous knock-in (KI) mice, which mimic ΔGAG mutation in the endogenous gene, exhibit motor deficits and deceased frequency of spontaneous excitatory post-synaptic currents (sEPSCs) and normal theta-burst-induced long-term potentiation (LTP) in the hippocampal CA1 region. Although *Dyt1* KI mice show decreased hippocampal torsinA levels, it is not clear whether the decreased torsinA level itself affects the synaptic plasticity or torsinA^ΔE^ does it. To analyze the effect of partial torsinA loss on motor behaviors and synaptic transmission, *Dyt1* heterozygous knock-out (KO) mice were examined as a model of a frame-shift DYT1 mutation in patients. Consistent with *Dyt1* KI mice, *Dyt1* heterozygous KO mice showed motor deficits in the beam-walking test. *Dyt1* heterozygous KO mice showed decreased hippocampal torsinA levels lower than those in *Dyt1* KI mice. Reduced sEPSCs and normal miniature excitatory post-synaptic currents (mEPSCs) were also observed in the acute hippocampal brain slices from *Dyt1* heterozygous KO mice, suggesting that the partial loss of torsinA function in *Dyt1* KI mice causes action potential-dependent neurotransmitter release deficits. On the other hand, *Dyt1* heterozygous KO mice showed enhanced hippocampal LTP, normal input-output relations and paired pulse ratios in the extracellular field recordings. The results suggest that maintaining an appropriate torsinA level is important to sustain normal motor performance, synaptic transmission and plasticity. Developing therapeutics to restore a normal torsinA level may help to prevent and treat the symptoms in DYT1 dystonia.

## Introduction

Dystonia is clinically defined as sustained muscle contractions that often involve both agonist and antagonist muscles, causing twisting and repetitive movements or abnormal postures [1, 2]. There are more than 20 different forms of monogenic dystonia, but only half have been linked to a specific gene [3]. The most common generalized, early-onset form of dystonia is DYT1 dystonia [Oppenheim’s dystonia; Online Mendelian Inheritance in Man (OMIM) identifier #128100, Dystonia 1]. DYT1 dystonia is inherited in an autosomal dominant fashion and has a reduced penetrance of 30% to 40% [4]. DYT1 dystonia is caused by mutations in *DYT1/TOR1A*, which codes for torsinA with 332 amino acids. Most of the patients have a trinucleotide deletion (ΔGAG) corresponding to a glutamic acid (torsinA^ΔE^) at 302 or 303 amino acid position in the carboxyl terminal region [5]. The ΔGAG mutation is related not only to generalized dystonia, but also to some forms of focal or multifocal dystonia [6, 7]. Homozygous *DYT1* ΔGAG mutation carriers have not been reported in humans. Although the ΔGAG mutation is the most common mutation, an 18 bp in-frame deletion mutation corresponding to 323–328 amino acid position [8–10], a 4 bp-deletion mutation resulting in a frame-shift at 311 amino acid position and a premature stop codon at 325 position [11], and an Arg288Gln missense mutation [12] have been reported. Whether torsinA^ΔE^ leads to loss-of-function, toxic-gain-of-function, or both remains unknown.

While many genetic animal models of DYT1 dystonia have been reported [13], the *Dyt1* ΔGAG heterozygous knock-in (KI) mouse is an ideal genetic mouse model for DYT1 dystonia with the ΔGAG mutation because it expresses the mutant allele from the endogenous promoter [14, 15]. TorsinA levels are reduced in *Dyt1* KI mouse brains similar to those in the human fibroblasts derived from a DYT1 dystonia patient, suggesting that ΔGAG mutation in the endogenous gene causes a partial loss of torsinA function in both humans and mice [15–17]. It appears that WT and mutant torsinA have different degradation pathways, *i.e*. WT torsinA is degraded through the proteasome pathway, while torsinA^ΔE^ is rapidly degraded via both the proteasome and lysosomal-autophagy pathways [18, 19].

Support for the toxic gain-of-function including dominant-negative-function of torsinA^ΔE^ largely come from over-expression models of the mutant protein. For example, overexpressing torsinA^ΔE^ resulted in a redistribution of torsinA^ΔE^ to the nuclear envelope in *Drosophila melanogaster* and various mammalian cell lines [20–26]. Redistributed torsinA^ΔE^ recruits wild-type (WT) torsinA from the endoplasmic reticulum [27, 28] and may lead to a loss-of-function of WT torsinA. TorsinA^ΔE^, but not WT torsinA, interacts with proteins involved in dopamine synthesis and storage in cultured cells [29, 30]. These studies suggest that the ΔGAG mutation may introduce toxic gain-of-function effects.

Recombinant human torsinA^ΔE^ produced by baculovirus expression system has ATPase activity *in vitro* with *k*cat and *K*m values similar to those of recombinant WT torsinA [21]. Another study showed an approximately 35% decrease of ATPase activity of recombinant human torsinA^ΔE^ produced in *Escherichia coli* [31]. On the other hand, recombinant mutant torsinA carrying the −18 bp mutation produced by *Escherichia coli* has an approximately 75% decrease of ATPase activity [31]. These studies suggest that ATPase activity is different between the mutation types.

DYT1 dystonia is a neuronal circuit disorder, rather than neurodegeneration disorder [32]. Electrophysiological recording in the hippocampal slice is one of the well-established experimental models to examine synaptic transmission and plasticity in the rodent brains and high quality recording data can be obtained with relative ease. The hippocampal CA3 pyramidal cells project to the CA1 pyramidal cells through Schaffer collaterals. Theta-burst stimulation in the CA3 region induces long-term potentiation (LTP) in the CA1 region. *Dyt1* KI mice exhibit normal theta-burst-induced LTP in the hippocampal CA1 region and no significant difference in input-output curves [33]. On the other hand, *Dyt1* KI mice exhibit enhanced pair pulse ratios (PPRs) in CA1 region. Consistent with PPR deficits, whole cell recording from the CA1 neurons showed a deceased frequency of sEPSC and normal frequency in mEPSC, suggesting action potential-dependent presynaptic neurotransmitter release deficits [34]. Although ΔGAG mutation may introduce loss-of-function, toxic gain-of-function including dominant-negative effects, or both, it is still not clear how each affects synaptic transmission and plasticity. *Dyt1* heterozygous KO mouse, derived from a frame-shift mutation by the deletion of exons 3–4, is one of the ideal models to analyze the effect of partial torsinA loss on motor and electrophysiological deficits due to the loss of one allele. It is also clinically relevant because the *Dyt1* heterozygous KO mouse mimics a frame-shift type *DYT1* mutation in DYT1 dystonia patients [11]. Although it was suggested that it will be critical to compare the behaviors of *Dyt1* heterozygous KI mice with *Dyt1* heterozygous KO mice [35], motor performance in *Dyt1* heterozygous KO mice has not been reported. Here, motor behavior, LTP, input-output curve, PPR, sEPSC and mEPSC were analyzed in *Dyt1* heterozygous KO mice as a model of partial loss of torsinA function.

## Results

### Motor deficits in *Dyt1* heterozygous KO mice

**Fig 1:**
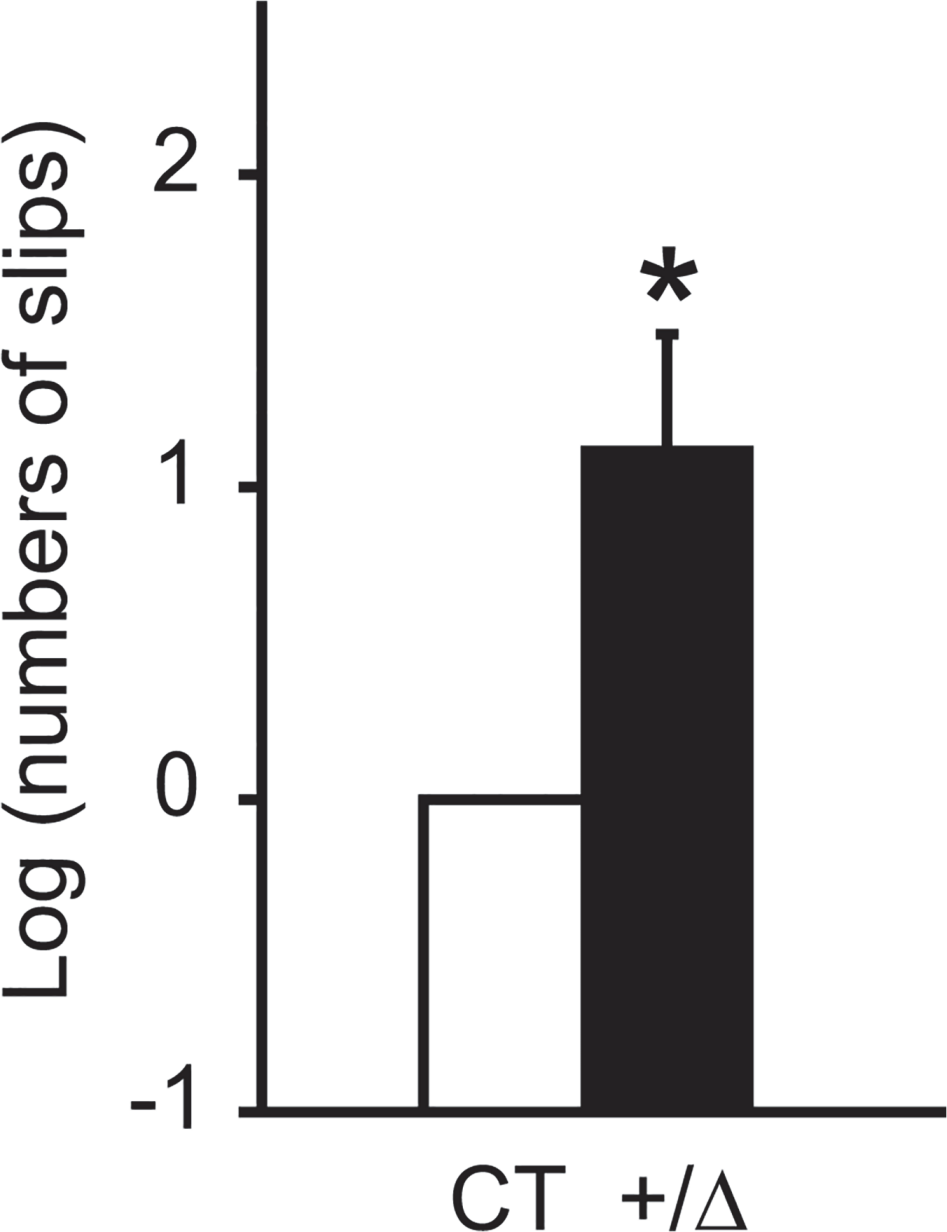
Beam-walking test in *Dyt1* heterozygous KO mice. *Dyt1* heterozygous KO mice (+/Δ) showed significantly increased numbers of slips compared to control mice (CT). The data were analyzed after log transformation to obtain a normal distribution. Control mice were normalized to zero. The vertical bars represent means ± standard errors. *P < 0.05.

*Dyt1* heterozygous KO mice were produced as previously described [36]. Contrast to *Dyt1* homozygous KO mice, *Dyt1* heterozygous KO mice can mature to adulthood. Behavioral semi-quantitative assessments of motor disorders did not show any significant alterations in hindpaw clasping, hindpaw dystonia, truncal dystonia and balance adjustments to a postural challenge. The results suggest that *Dyt1* heterozygous KO mice did not exhibit overt dystonic symptoms. Motor coordination and balance were further analyzed by the beam-walking test. Mice were trained to transverse a medium square beam for two days and the trained mice were tested twice on four different beams. The numbers of hindpaw slips were counted and compared between *Dyt1* heterozygous KO mice and control mice. *Dyt1* heterozygous KO mice showed 205% more slip numbers in the beam-walking test (Fig. 1; *p* = 0.037), suggesting motor deficits. The results suggest that the decreased torsinA function resulting from a single null allele is sufficient to exhibit motor deficits. In an accelerated rotarod test, each mouse was put on an accelerated rotarod and the latency to fall was measured. *Dyt1* heterozygous KO mice did not show a significant difference in latency to fall (*p* = 0.3, data not shown). Since mice can hold onto the rotarod with four paws and the latency to fall is an indicator of total motor performance, the results suggest no significant motor symptoms in total motor performance with four paws.

### Reduction of striatal and hippocampal torsinA levels in *Dyt1* heterozygous KO mice

**Fig 2:**
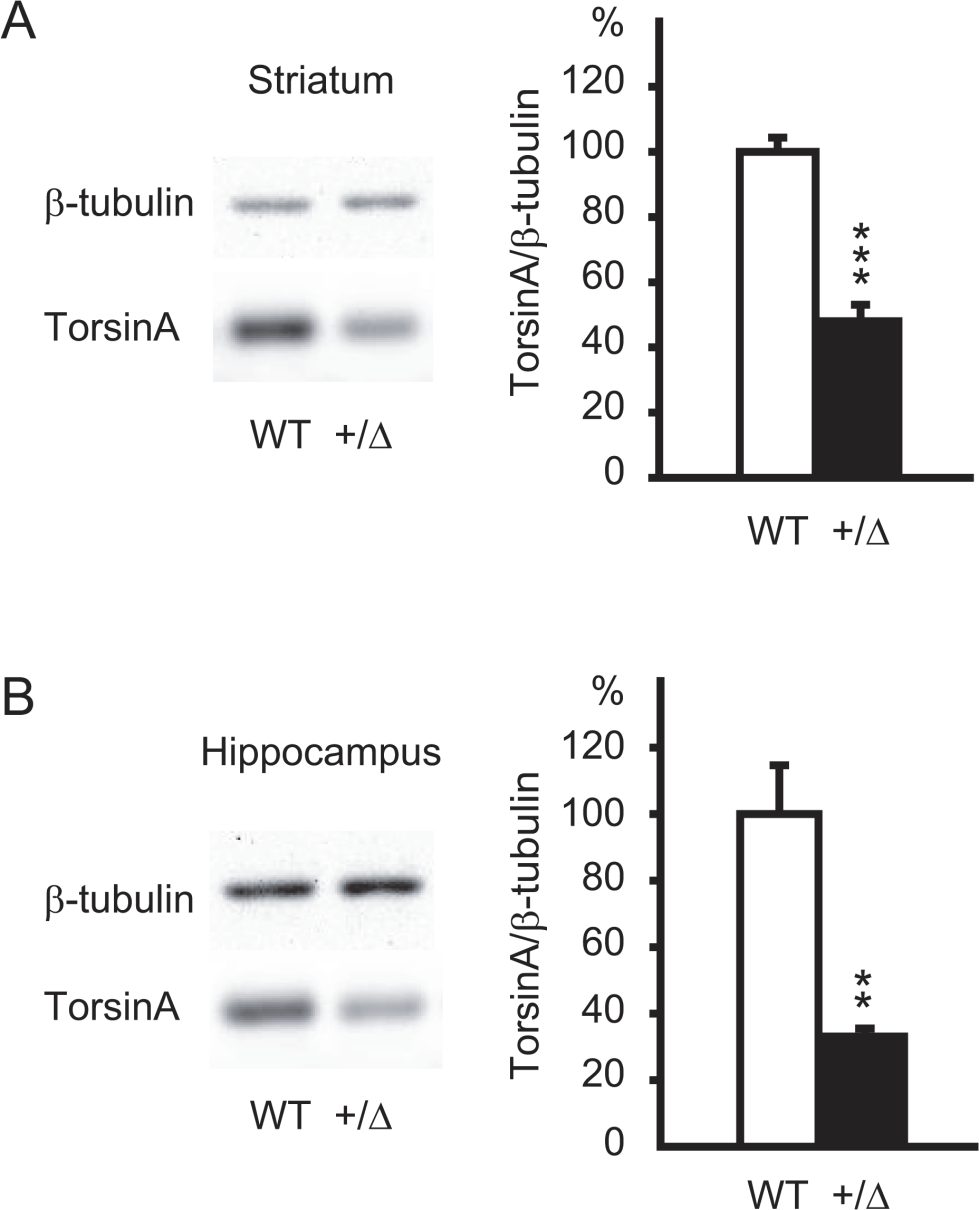
Reduced striatal and hippocampal torsinA levels in *Dyt1* heterozygous KO mice. The striatal (A) and hippocampal (B) torsinA levels were compared between wild-type (WT) and *Dyt1* heterozygous KO (+/Δ) mice by western blot. Representative images of western blot (left) and the quantified torsinA levels (right) are shown for each case. The torsinA levels were normalized to β-tubulin and the vertical bars represent means ± standard errors. **p < 0.01, ***p < 0.001.

*Dyt1* KI mice show reduced striatal torsinA levels (WT, 100.0 ± 10.3%, n = 4; KI, 59.4 ± 8.4%, n = 4; *p* = 0.02) [16] and loss of striatal torsinA function alone is sufficient to show beam-walking deficits similar to *Dyt1* KI mice [37]. To examine the striatal torsinA levels in *Dyt1* heterozygous KO mice, the striata were dissected and the torsinA levels were compared between *Dyt1* heterozygous KO mice and the WT littermates by Western blot analysis. *Dyt1* heterozygous KO mice showed significant reduction of the striatal torsinA levels (WT, 100.0 ± 4.4%, n = 4; heterozygous KO, 48.0 ± 5.1%, n = 4; *p* = 0.0002; Fig. 2A).

Since the acute hippocampal slices were used in the following electrophysiological experiments to examine synaptic transmission and plasticity, the hippocampal torsinA levels were also quantified in *Dyt1* heterozygous KO mice. The hippocampi were dissected from the same *Dyt1* heterozygous KO mice and the WT littermates and the torsinA levels were compared by Western blot analysis. *Dyt1* heterozygous KO mice also showed significant reductions of the hippocampal torsinA levels (WT, 100.0 ± 14.7%, n = 4; heterozygous KO, 33.1 ± 2.4%, n = 4; *p* = 0.004; Fig. 2B). The hippocampal torsinA levels in *Dyt1* heterozygous KO mice were reduced more than those in KI mice (KI, 66.4 ± 8.6%, n = 3; WT, 100 ± 7.5%, n = 3; *p* = 0.04), which has been reported in a previous paper [34].

### Decreased frequency of sEPSCs in *Dyt1* heterozygous KO mice

**Fig 3:**
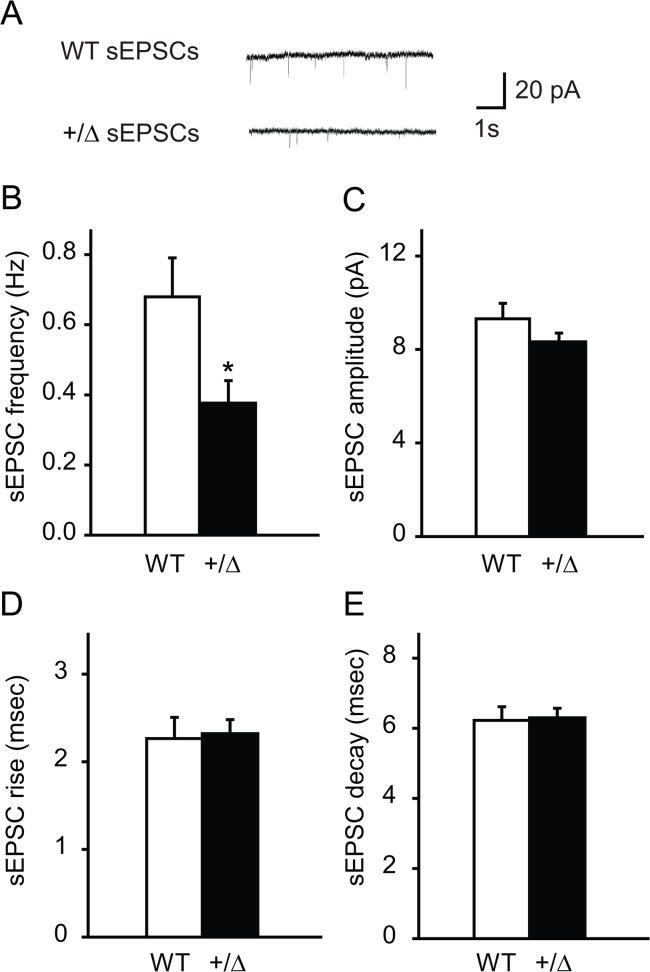
Decreased frequency of sEPSCs in *Dyt1* heterozygous KO mice. (A) Representative traces for sEPSCs. *Dyt1* heterozygous KO mice had a significantly decreased frequency of sEPSCs (B), but no change in either the amplitude (C), or rise (D) and decay (E) times of these events. The vertical bars represent means ± standard errors. *p<0.05.

Since evoked and spontaneous neurotransmitter releases are differentially regulated at central synapses [38], spontaneous excitatory post-synaptic currents (sEPSCs) from the CA1 pyramidal cells were first analyzed by a whole-cell voltage-clamp under a GABA_A_ receptor blocker in *Dyt1* heterozygous KO mice and WT littermates (Fig. 3A). The frequency of sEPSCs was significantly reduced in *Dyt1* heterozygous KO mice compared to that in WT littermates (Fig. 3B; WT, 0.68 ± 0.11; KO, 0.38 ± 0.06; *p* = 0.02). On the other hand, there were no significant changes in the amplitude (Fig. 3C; WT, 9.3 ± 0.7; KO, 8.3 ± 0.4; *p* = 0.19) and rise (Fig. 3D; WT, 2.3 ± 0.2; KO, 2.3 ± 0.2; *p* = 0.84) and decay times (Fig. 3E; WT, 6.2 ± 0.4; KO, 6.3 ± 0.3; *p* = 0.87) of sEPSC between *Dyt1* heterozygous KO mice and WT littermates.

### Normal mEPSCs in *Dyt1* heterozygous KO mice

**Fig 4:**
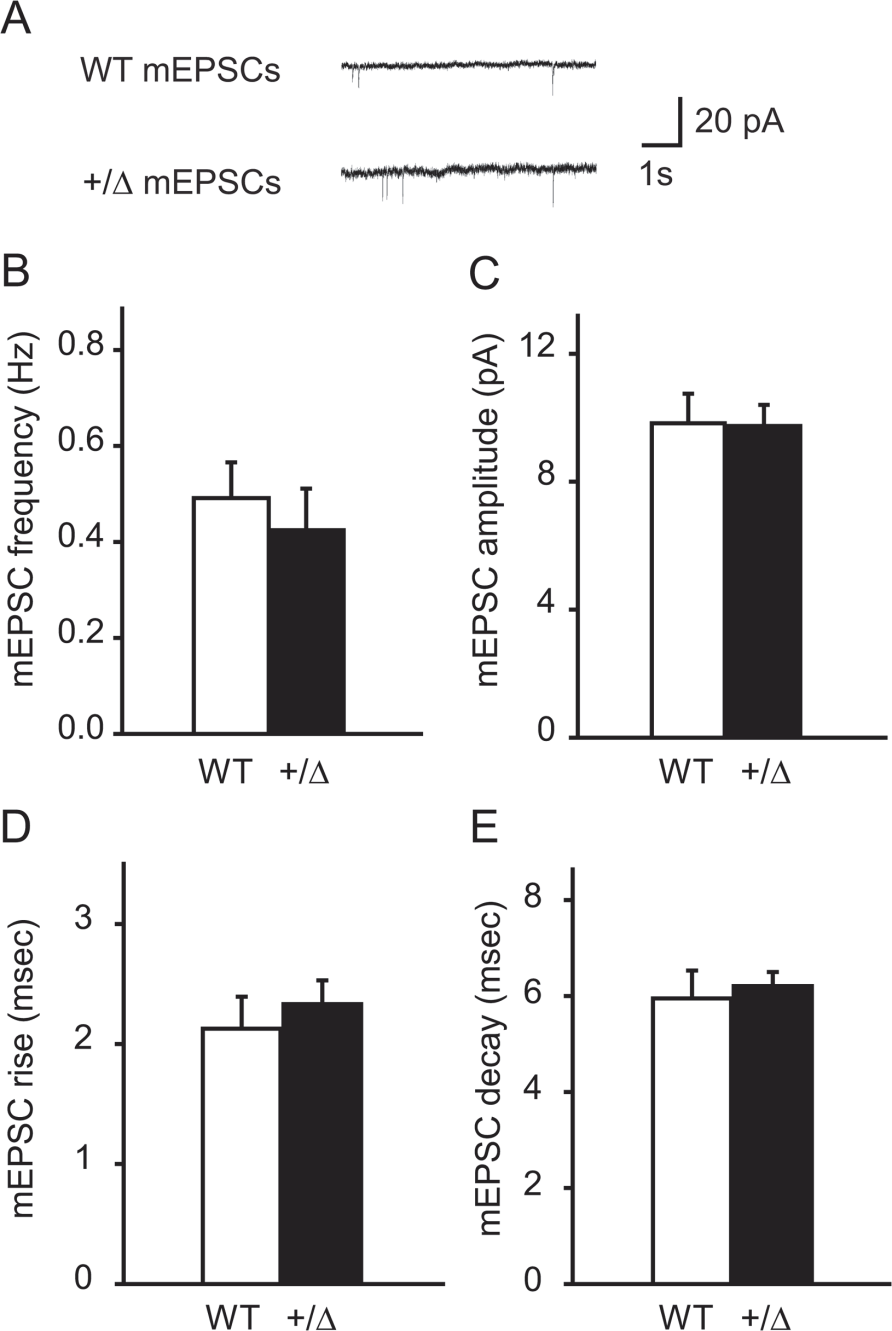
Normal mEPSCs in *Dyt1* heterozygous KO mice. (A) Representative traces for mEPSCs. *Dyt1* heterozygous KO mice had no change in frequency (B), amplitude (C), or rise (D) and decay (E) times of mEPSCs. The vertical bars represent means ± standard errors.

Since sEPSCs are the mixture of signals derived from action potential-dependent and independent transmitter releases, only action potential-independent spontaneous EPSCs were further analyzed by blocking voltage-dependent sodium channels in addition to blocking GABA_A_ receptors in *Dyt1* heterozygous KO mice and WT littermates (Fig. 4A). There were no significant changes in the frequency (Fig. 4B; WT, 0.49 ± 0.07; KO, 0.42 ± 0.09; *p* = 0.58), amplitude (Fig. 4C; WT, 9.8 ± 0.9; KO, 9.7 ± 0.7; *p* = 0.94), rise (Fig. 4D; WT, 2.1 ± 0.3; KO, 2.3 ± 0.2; *p* = 0.53) and decay times (Fig. 4E; WT, 6.0 ± 0.6; KO, 6.2 ± 0.3; *p* = 0.67) of mEPSC in *Dyt1* heterozygous KO mice compared to WT mice. The mEPSCs data suggest that the action potential-independent spontaneous pre-synaptic release was normal.

On the other hand, there was a significant decrease in frequency of sEPSCs in *Dyt1* heterozygous KO mice (Fig. 3B); these results suggest that the action potential-dependent pre-synaptic release was impaired in *Dyt1* heterozygous KO mice. These results were similar to *Dyt1* KI mice as previously reported [34].

### Enhanced hippocampal CA1 LTP in *Dyt1* heterozygous KO mice

**Fig 5:**
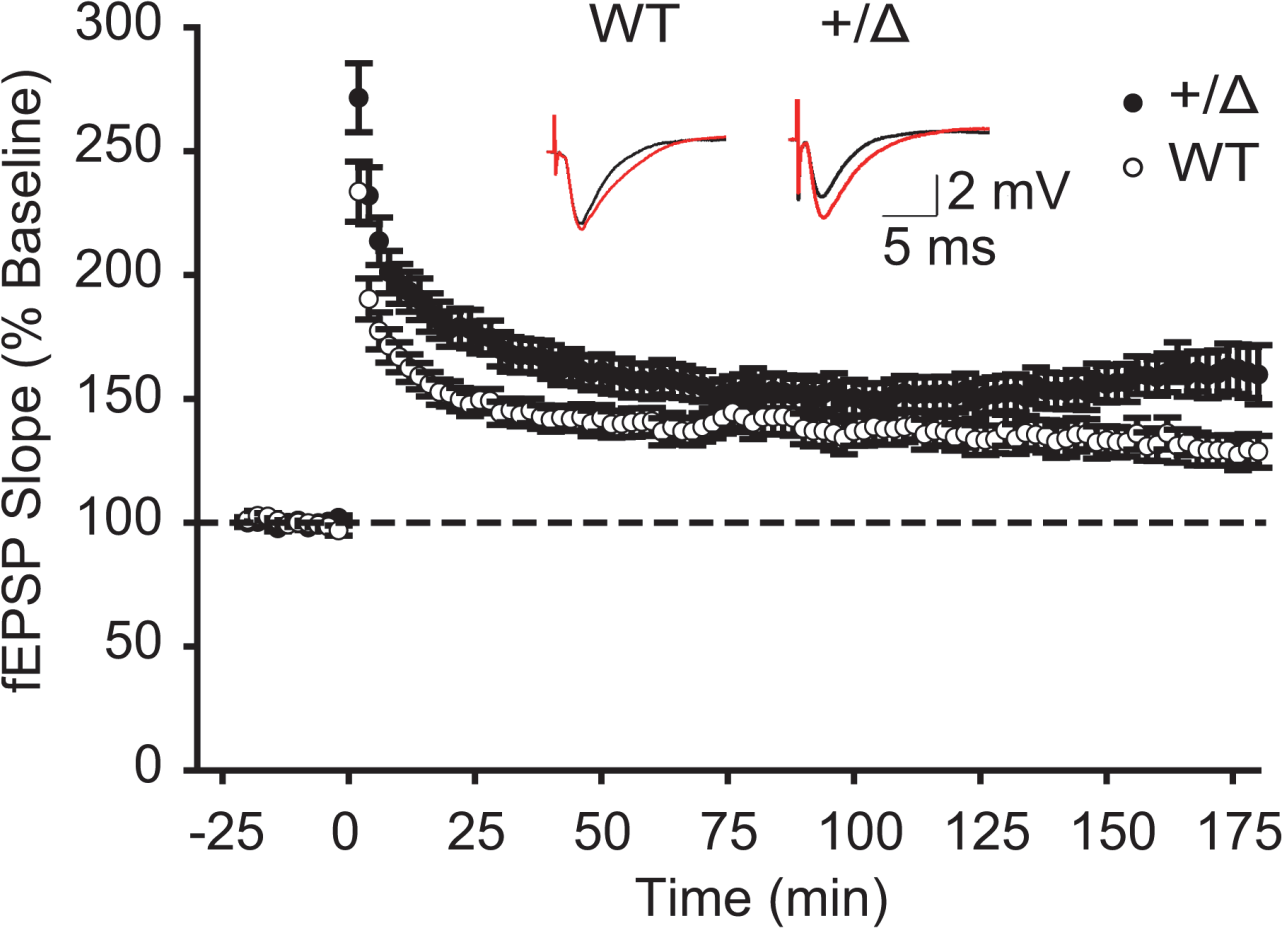
Enhance hippocampal CA1 LTP in *Dyt1* heterozygous KO mice. Compared to their control mice, there was a significantly enhanced LTP in *Dyt1* heterozygous KO (+/Δ) mice. The dashed lines indicate the mean basal synaptic responses and time 0 min indicate the induction of LTP by theta burst stimulation. Inset shows representative traces 4 min before (black traces) and 2hr after LTP (red). Average results from control and the mutant slices were plotted (means ± errors). Open circles represent data from WT littermates. Filled circles represent data from the *Dyt1* heterozygous KO mice.

Extracellular field-recording of the Schaffer collaterals pathway in the acute hippocampal slice is one of the established methods to examine synaptic transmission and plasticity. Theta-burst stimulations in the hippocampal CA3 region induce LTP in the CA1 region. In the previous studies, *Dyt1* KI mice show no significant change of CA1 LTP in comparison to WT littermates [33, 39]. Here, CA1 LTP was measured in *Dyt1* heterozygous KO mice and their WT littermates. Unlike *Dyt1* KI mice, *Dyt1* heterozygous KO mice had significantly enhanced CA1 LTP compared to their WT littermates (WT, 132 ± 6%, 29 slices from 5 mice; +/Δ, 153 ± 5.7%, 28 slices from 6 mice, *p* < 0.05; Fig. 5). These results suggest that the decreased torsinA level, lower than those in *Dyt1* KI mice, produces enhanced LTP.

### No significant difference in input-output curves in *Dyt1* heterozygous KO mice

**Fig 6:**
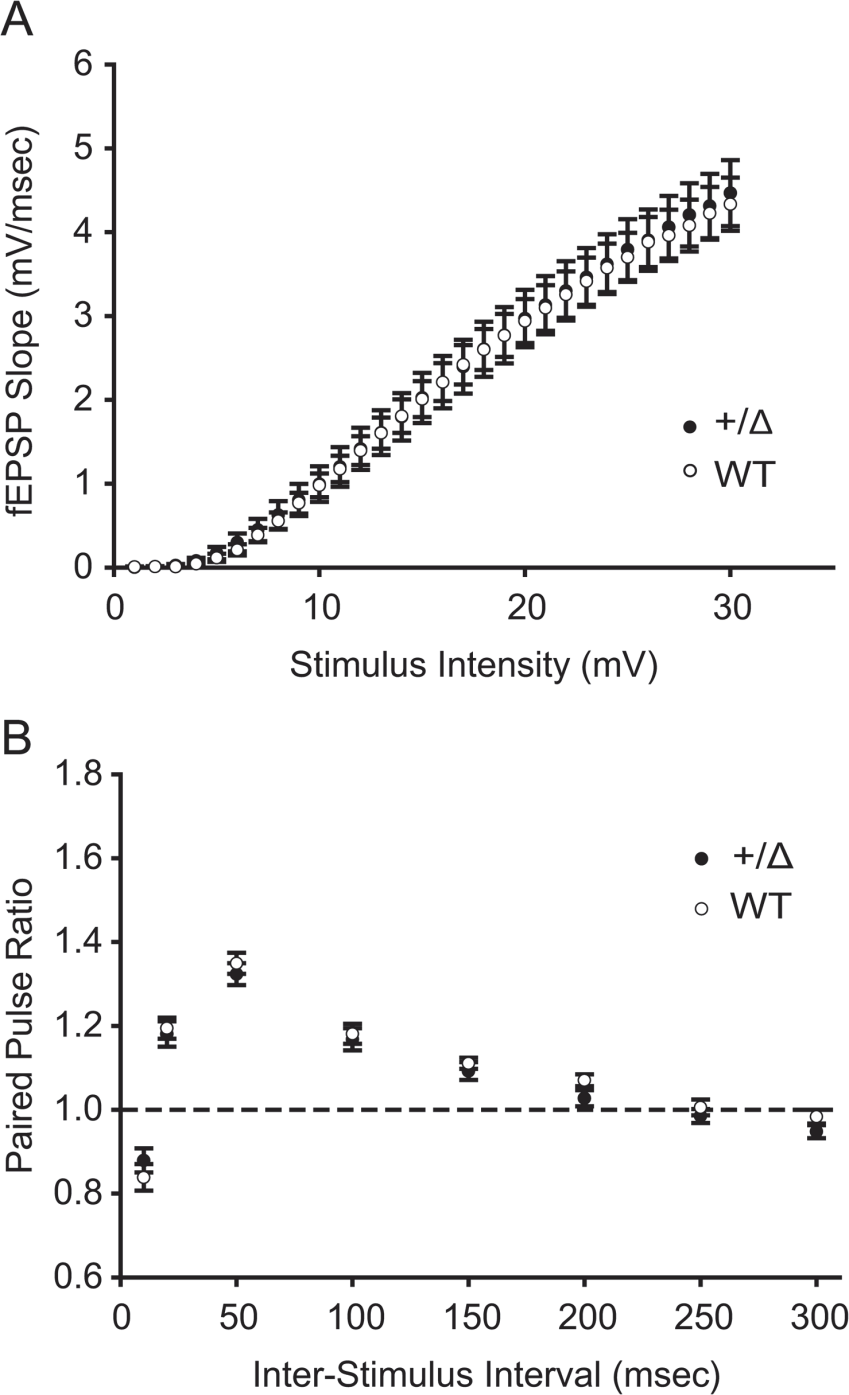
Normal input-output curves and paired pulse ratios (PPRs) in *Dyt1* heterozygous KO mice. The input-output curves in *Dyt1* heterozygous KO (+/Δ) mice were unaffected (A). Overall PPRs in *Dyt1* heterozygous KO (+/Δ) mice were not altered (B). Open circles represent data from WT littermates. Filled circles represent data from *Dyt1* heterozygous KO mice. Circles and bars represent means ± standard errors.

To determine whether the stimulus intensity-dependent basal synaptic transmission is altered in *Dyt1* heterozygous KO mice, input-output curves were obtained by measuring the post-synaptic potential slope with varying stimulus intensities. *Dyt1* heterozygous KO mice showed no significant change in the input-output curve compared to WT littermates (Fig. 6A; *p* = 1.00). The present results suggest that there is no significant change in the overall base line of theta burst-induced synaptic transmissions of *Dyt1* heterozygous KO mice, which is similar to the normal input-output curve reported in *Dyt1* KI mice [34, 39].

### No significant difference in PPRs

Extracellular field-recordings at various inter-stimulus intervals are commonly used to analyze the probability of synaptic vesicle release. When two consecutive action potentials are elicited in the presynaptic neurons, the amplitude of the second post-synaptic currents (EPSC) is inversely related to the amplitude of the first. Paired-pulse facilitation is observed when the first EPSC is smaller. On the other hand, paired-pulse depression is observed when the first EPSC is larger. The PPRs are inversely proportional to the probability of synaptic vesicle release [40]. A previous study showed enhanced PPRs in *Dyt1* KI mice, suggesting pre-synaptic release deficits. To examine whether the enhanced PPRs are caused by loss of torsinA function, PPRs were examined in *Dyt1*heterozygous KO mice. Overall, *Dyt1* heterozygous KO mice showed normal PPRs in comparison to WT littermates (Fig. 6B).

## Discussion

Consistent with *Dyt1* KI [14] and *Dyt1* homozygous knock-down (KD) mice [41], *Dyt1* heterozygous KO mice showed motor deficits in the beam-walking test. This result suggests that partial loss of torsinA function derived from a single null allele is sufficient to exhibit motor deficits similar to *Dyt1* KI and *Dyt1* homozygous KD mice. *Dyt1* heterozygous KO mice expressed reduced hippocampal torsinA levels, which were lower than those in *Dyt1* KI mice [34]. Whole-cell recordings of sEPSCs and mEPSCs from CA1 neurons revealed that *Dyt1* heterozygous KO mice had action potential-dependent neurotransmitter release deficits similar to those in *Dyt1* KI mice, suggesting that partial loss of torsinA function itself causes the synaptic transmission deficits. On the other hand, *Dyt1* heterozygous KO mice showed enhanced CA1 LTP in contrast with normal CA1 LTP in *Dyt1* KI mice [33]. The enhanced LTP in *Dyt1* heterozygous KO mice are likely caused by lower torsinA levels. The results suggest that significant reduction of torsinA levels causes a profound deficit in synaptic plasticity. Overall, the current results suggest that maintaining an appropriate torsinA level is important to sustain normal motor performance, synaptic transmission and plasticity. Developing therapeutics to restore a normal torsinA level may help to prevent and treat the symptoms in DYT1 dystonia.

*Dyt1* heterozygous KO mice showed reduced torsinA levels and motor deficits in beam-walking. Since *Dyt1* KI mice also show reduced torsinA level [16, 34] and motor deficits in beam-walking [14], the motor deficits found in *Dyt1* KI mice may be caused by a partial loss of torsinA function derived from the single ΔGAG allele. The beam-walking deficits are commonly observed in genetic dystonia rodent models, such as knock-in [14], knock-down [41], conditional KO [17, 36, 37], and transgenic [42–44] models of DYT1 dystonia; KO [45] and conditional KO [46, 47] models of DYT11 dystonia, and DYT12 dystonia model [48], suggesting that beam-walking is an excellent behavioral test to evaluate their motor symptoms in genetic dystonia mouse models.

Since *Dyt1* KI mice show action potential-dependent pre-synaptic release deficits in the hippocampal acute slices, here synaptic transmission was measured in *Dyt1* heterozygous KO mice to examine whether the release deficits in *Dyt1* KI mice are caused by a partial loss of torsinA function. *Dyt1* heterozygous KO mice showed decreased frequency in sEPSCs and normal mEPSCs similar to those in *Dyt1* KI mice [34]. On the other hand, *Dyt1* heterozygous KO mice exhibited normal PPRs and *Dyt1* KI mice show enhanced PPRs. The reason why these differences appeared is not known. Although *Dyt1* KI mice show enhanced PPRs, the enhancements appeared only in two out of eight inter-event intervals examined. Since *Dyt1* KI mice show normal PPRs in other six inter-event intervals similar to *Dyt1* heterozygous KO mice, the discrepancy of PPRs between *Dyt1* KI mice and *Dyt1* heterozygous KO mice are relatively small. Overall, the results suggest that the action potential-dependent pre-synaptic release deficits in *Dyt1* KI mice may be caused by a partial loss of torsinA function.

The molecular mechanism of action potential-dependent presynaptic release deficits in *Dyt1* heterozygous KO and KI mice is not known. One possible scenario could involve interaction between torsinA and snapin [49, 50]. Snapin is one of the SNAP-25 binding proteins and is implicated in synaptic transmission [51], such as synchronization of synaptic vesicle fusion [52]. TorsinA binds to CSN4 and snapin and regulates synaptic release in neuroblastoma cells [53] and modulates synaptic vesicle recycling in other cultured cell lines [54]. Moreover, snapin is expressed in the hippocampal pyramidal neurons and regulates type VI adenylyl cyclase [55]. Therefore, partial loss of torsinA function may affect snapin functions not only for neurotransmitter releasing pathway, but also regulation of cAMP synthesis in the neurons. Recently, altered cAMP level and response have been reported in DYT1 animal model and patient cell lines [56]. Further investigation of the snapin pathways in *Dyt1* mutant mice may be one of the important future studies.

*Dyt1* heterozygous KO mice showed normal mEPSCs similar to those in *Dyt1* KI mice [34]. These mEPSCs were recorded in CA1 pyramidal neurons of the acute brain slices derived from juvenile mice by blocking voltage-dependent sodium channels (TTX) in addition to blocking GABA_A_ receptors. On the other hand, in the presence of TTX and absence of GABA_A_ receptor antagonists, increased miniature release events were observed in cultured hippocampal neurons derived from a different line of *Dyt1* knockin mice [57]. Although the reason why different results were obtained is not clear, it should be noted that each recording was performed under quite different conditions. CA1 pyramidal neurons in the acute brain slices still maintain intrinsic synaptic circuits of CA3 pyramidal neurons through Schaffer collaterals. On the other hand, the cultured neurons were derived from the CA3-CA1 regions dissected from the hippocampus in neonatal mice at postnatal days 0–1. The cells were seeded with a rat glial feeder layers and cultured for 11–14 days. The differences in developmental and recording conditions may account for the discrepancy between the results.

*Dyt1* heterozygous KO mice had significantly enhanced CA1 LTP, while *Dyt1* KI mice show no significant change of CA1 LTP [33, 39]. These results suggest that the decreased torsinA level, lower than those in *Dyt1* KI mice, produces enhanced LTP. There seems to be a threshold between 33.1 and 66.4% of torsinA levels to exhibit enhanced CA1 LTP. The reduction of torsinA in *Dyt1* KI mice may not be sufficient to enhance CA1 LTP. Since the hippocampus contribute to spatial memory rather than motor performance, the pre-synaptic release deficits and enhanced LTP should not contribute directly to dystonia symptoms. However, the present results suggest that the partial loss of torsinA function mimics the effect of ΔGAG mutation on both behavioral and electrophysiological outputs. Recent findings showed an impaired corticostriatal LTD and its recovery by anticholinergics in *Dyt1* KI mice [58, 59]. Partial loss of torsinA function derived from a ΔGAG mutation allele may contribute to the electrophysiological alterations in other brain regions and pathophysiology of DYT1 dystonia.

## Materials and Methods

### Mice

All experiments were carried out in compliance with the USPHS Guide for Care and Use of Laboratory Animals and approved by the IACUC at the University of Illinois at Urbana-Champaign, University of Alabama at Birmingham, and University of Florida. *Dyt1* heterozygous KO mice were produced as previously described [36]. For the recording and Western blot experiments, only male mice were used to avoid the effect of estrus cycle in female mice. All mice were housed under a 12-hour light 12-hour dark cycle with *ad libitum* access to food and water.

### Behavioral semi-quantitative assessments of motor disorders

Motor behaviors were assessed by semi-quantitative assessments, beam-walking and accelerated rotarod tests in this order with over one week intervals between each test. Behavioral semi-quantitative assessments of motor disorders were performed as described earlier [14, 60]. *Dyt1* heterozygous KO (female, n = 9; male, n = 8) and control (female, n = 12; male, n = 13) mice from 3–7 months old were placed on a table and assessments of hindpaw clasping, hindpaw dystonia, truncal dystonia and balance adjustments to a postural challenge were performed. The hindpaw clasping was assessed as hindpaw movements for postural adjustment and attempt to straighten up while the mouse was suspended by the mid-tail. The hindpaw dystonia was assessed as the increased spacing between the limbs, poor limb coordination, crouching posture and impairment of gait. Truncal dystonia was assessed as the flexed posture. Postural challenge was performed by flipping the mouse onto its back and the ease of righting was noted.

### Beam-walking test

*Dyt1* heterozygous KO and control mice were used for beam-walking test as described earlier [14]. Briefly, the beam-walking test was performed within the last 8 hours of the light period after acclimation to a sound-attenuated testing room for 1 hour. Mice were trained to transverse a medium square beam (14 mm wide) in three consecutive trials each day for 2 days and tested twice each on the medium square beam and a medium round beam (17mm diameter) on the third day. The mice were then tested twice each on a small round beam (10 mm diameter) and a small square beam (7 mm wide) on the fourth day. Slips number of the hind paw on each side during transverse on the 80 cm-length beams were counted by investigators blind to the genotypes.

### Accelerated rotarod test

The motor performance was further assessed with Economex accelerating rotarod (Columbus Instruments) as described earlier [14]. The apparatus started at an initial speed of 4 rpm. Rod speed was gradually accelerated at a rate of 0.2 rpm/s. The latency to fall was measured with a cutoff time of 2 min. *Dyt1* heterozygous KO and control mice were tested for three trials on each day for 2 days. The trials within the same day were performed at approximately 1 hour intervals.

### Western blot for torsinA

The striata and hippocampi were dissected from 4 heterozygous KO, 4 WT littermates at 2 months old and quickly frozen in liquid nitrogen. The striata and hippocampi were homogenized in 200 μl and 400 μl of ice-cold lysis buffer [50 mM Tris·Cl (pH 7.4), 175 mM NaCl, 5 mM EDTA∙2Na, complete Mini (Roche)], respectively, and sonicated for 10 sec. One ninth volume of 10% Triton X-100 in lysis buffer was added to the homogenates. The homogenates were incubated on ice for 30 min, and then centrifuged at 10,000×*g* for 15 min at 4°C. The supernatants were then collected and the protein concentration was measured by Bradford assay with bovine serum albumin as standards [61]. The homogenates were mixed with sodium dodecyl sulfate polyacrylamide gel electrophoresis (SDS-PAGE) loading buffer and boiled for 5 min, incubated on ice for 1 min, and then centrifuged for 5 min to obtain the supernatant. Forty μg of total protein was separated by SDS-PAGE and transferred to a PROTRAN nitrocellulose transfer membrane (Whatman). The membrane was blocked in 5% milk in TBS-T buffer [20 mM Tris·Cl (pH 7.6), 137 mM NaCl, 0.1% (v/v) Tween 20] for 1 hour at room temperature. The membrane was cut into two and probed for torsinA and β-tubulin separately. Each membrane was either incubated in the rabbit anti-torsinA (Abcam; ab34540; 1:1,000 dilution) or HRP-conjugated anti-β-tubulin (Santa Cruz; sc-5274 HRP; 1:250 dilution) in blocking buffer [TBS-T containing 5% milk (Bio-rad)] overnight at 4°C. The high specificity of the anti-torsinA has already been confirmed by Western blot using *Dyt1* KO mouse striatum protein extract as previously described [16]. After overnight incubation with the primary antibody, the membranes for torsinA were washed in TBS-T buffer for 10 min three times, incubated with bovine anti-rabbit IgG-HRP (Santa Cruz; sc-2370; 1:2,000 dilution) in the blocking buffer at room temperature for 1 hour, and washed for 10 min each nine times. On the other hand, the membranes incubated with HRP-conjugated anti-β-tubulin were washed in TBS-T buffer for 10 min each nine times. The bands were detected by SuperSignal West Pico Chemiluminescent Substrate (Thermo Scientific) and the signals were captured by an Alpha Innotech FluorChem FC2. The density of each band was quantified with UN-SCAN-IT gel software (Silk Scientific). Molecular masses were estimated from the migration of Precision Plus Protein Standards All Blue marker (Bio-rad). The density of torsinA band was normalized to that of β-tubulin. Western blot analysis was performed in triplicate. The average torsinA levels in WT mice were normalized to 100% for those in *Dyt1* heterozygous KO mice.

### Whole-cell recordings

Recordings were conducted from 5 juvenile *Dyt1* heterozygous KO and 5 WT littermate mice (11–21 days old). Animals were anesthetized by the inhalation of isoflurane, decapitated and the brains were rapidly removed. 350 μm-thick coronal brain slices were cut with a Vibratome (Technical Products International, St. Louis, MO). Slices were first incubated in artificial cerebrospinal fluid (ACSF) containing (in mM) 124 NaCl, 26 NaHCO_2_, 1.25 NaH_2_PO_4_, 2.5 KCl, 1 CaCl_2_, 6 MgCl_2_, 10 D-glucose gassed with 95% O_2_ and 5% CO_2_ at 35°C for 30 min, then continued to incubate at room temperature (22°C). After at least 1 hr incubation, a slice was transferred to a submerged recording chamber with continuous flow (2 ml/min) of ACSF as described above except for 2 mM CaCl_2_ and 2 mM MgCl_2_ and gassed with 95% O_2_–5% CO_2_ giving pH 7.4. All experiments were carried out at 32°C to 33°C. Whole-cell recordings were made from pyramidal cells in the hippocampal CA1 region using infrared differential interference contrast microscopy and an Axopatch 1Damplifier (Axon Instruments, Foster City, CA). Patch electrodes had a resistance of 3–5 MΩ when filled with intracellular solution containing (in mM): 125 K-gluconate, 8 NaCl, 10 HEPES, 2 MgATP, 0.3 Na_3_GTP, 0.2 EGTA, and 0.1% biocytin (pH 7.3 with KOH, osmolarity 290–300 mOsM). sEPSCs and mEPSCs were recorded at a holding potential of −70 mV and at the presence of 50μM picrotoxin which blocked GABAergic synaptic activity. 1 μM TTX was also applied to bath solution for mEPSCs recordings, which blocked the transmitter release driven by action potentials. Series resistance was 15–20 MΩ and cells were rejected if it changed more than 20% throughout the recording. All drugs were purchased from Sigma-Aldrich. Data were acquired using pClamp 10 software. The recordings were started 5 min after accessing cells to allow for stabilization of spontaneous synaptic activity. Analysis of sEPSCs and mEPSCs was based on 5 min continuous recordings from each cell. Synaptic events were detected using the Mini Analysis Program (Synaptosoft) with parameters optimized for each cell and then visually confirmed prior to analysis. The peak amplitude, 10–90% rise time and the decay time constant were measured based on the average of all events aligned by rise phase. The electrophysiological recordings were performed by investigators blind to the genotypes.

### Preparation of the hippocampal slices for extracellular field recordings

Extracellular field recordings were performed in 4 adult *Dyt1* heterozygous KO mice and 6 matched WT littermate mice as previously described [34, 62]. Hippocampi were rapidly removed from these adult mice and briefly chilled in ice-cold cutting saline (110 mM sucrose, 60 mM NaCl, 3 mM KCl, 1.25 mM NaH_2_PO_4_, 28 mM NaHCO_3_, 5 mM D-glucose, 500 μM CaCl_2_, 7 mM MgCl_2_, and 600 μM ascorbate). Transverse slices 400-μm thick were prepared with a Vibratome and maintained at least 45 min in a holding chamber containing 50% artificial cerebral spinal fluid (aCSF; 125 mM NaCl, 2.5 mM KCl, 1.25 mM NaH_2_PO_4_, 25 mM NaHCO_3_, 25 mM D-glucose, 2 mM CaCl_2_, and 1 mM MgCl_2_) and 50% cutting saline. The slices were then transferred to a recording chamber and perfused (1 ml/min) with 100% aCSF. Slices were allowed to equilibrate for 60–90 min in a Fine Science Tools interface chamber at 30°C. All solutions were continuously bubbled with 95% O_2_/5% CO_2_.

### Set-up and electrode placement

For extracellular field recordings, glass recording electrodes were pulled from capillary glass tubes using a horizontal electrode puller (Narishige), and filled with aCSF. The input resistance of each electrode was tested by applying a current pulse and breaking the tip until a resistance of 1–3 MΩ was obtained. Recording electrodes were placed in stratum radiatum of hippocampal area CA1. Test stimuli were delivered to the Schaffer collateral/commissural pathway with bipolar Teflon coated platinum stimulating electrode positioned in the stratum radiatum of area CA3. Responses were recorded through a personal computer using AxoClamp pClamp8 data acquisition software. Excitatory Post-Synaptic Potential (EPSP) slope measurements were taken after the fiber volley to eliminate contamination by population spikes.

### Long-term potentiation

Followed by at least 20 min of stable baseline recording, LTP was induced with two, 100 Hz tetani (1 sec), with an interval of 20 sec between tetani. Synaptic efficacy was monitored by recording field EPSPs (fEPSPs) every 20 sec beginning 0.5 hour prior to and ending 3 hours after the induction of LTP (traces were averaged for every 2 min interval). For statistical comparison, all traces were averaged over 2 min intervals from 36 min to 176 min, when a majority of recordings ceased. The first 35 min were excluded to prevent averaging the post-tetanic potentiation.

### Input-output curves

Test stimuli were delivered and responses were recorded at 0.05 Hz; every six consecutive responses over a 2 min period were pooled and averaged. fEPSPs were recorded in response to increasing intensities of stimulation (1 mV-30 mV).

### Paired pulse ratio

PPRs were measured at various inter-stimulus intervals (10, 20, 50, 100, 150, 200, 250, and 300 msec). All experimental stimuli were set to an intensity that evoked 50% of the maximum fEPSP slope.

### Statistics

The beam-walking test data were analyzed by logistic regression (GENMOD) with negative binominal distribution using GEE model in SAS/STAT Analyst software (SAS institute Inc. NC), using sex, age and body weight as variables [36]. The data were analyzed after log transformation to obtain a normal distribution. Control mice were normalized to zero. The hippocampal torsinA levels were compared between *Dyt1* heterozygous KO and their littermate WT mice using Student’s t-test. The sEPSC and mEPSC data were analyzed using Student’s t-test. The fEPSP slope data were analyzed by the Kolmogorov-Smirnov test in SAS/STAT Analyst software (SAS Institute Inc. NC). LTP data were analyzed by ANOVA mixed model with repeated measurements in the software. The data for PPRs were analyzed either for data at each inter-event interval or all data regardless of the inter-event intervals. Significance was assigned at *p* < 0.05.
