# Correction: Behavioral and Electrophysiological Characterization of *Dyt1* Heterozygous Knockout Mice

**DOI:** 10.1371/journal.pone.0126539

**Published:** 2015-04-15

**Authors:** The PLOS ONE Staff

The following information is missing from the Funding section: This study was supported by the Bachmann-Strauss Dystonia and Parkinson Foundation, Inc.

The complete, correct funding information is as follows: This work was supported by Tyler’s Hope for a Dystonia Cure, Inc., Dystonia Medical Research Foundation, Bachmann-Strauss Dystonia and Parkinson Foundation, Inc., National Institutes of Health grants (NS37409, NS47466, NS47692, NS54246, NS57098, NS65273, NS72872, NS74423, and NS82244), Howard Hughes Med-Grad Graduate Fellowship (CCC), and startup funds from the Lucille P. Markey Charitable Trust (UIUC) and Department of Neurology (UAB and UF). The funders had no role in either the study design, data collection and analysis, decision to publish, or preparation of the manuscript.

The publisher apologizes for the error.

## References

[1] Yokoi F, Chen H-X, Dang MT, Cheetham CC, Campbell SL, Roper SN, et al (2015) Behavioral and Electrophysiological Characterization of *Dyt1* Heterozygous Knockout Mice. PLoS ONE 10(3): e0120916 doi: 10.1371/journal.pone.0120916 25799505 10.1371/journal.pone.0120916 PMC4370625

